# Supplementary material for: Poly(alizarin red S) modified glassy carbon electrode for square wave adsorptive stripping voltammetric determination of metronidazole in tablet formulation
Source: PLoS One. 2020 Dec 22;15(12):e0244115. doi: 10.1371/journal.pone.0244115 (PMC7755201; doi:10.1371/journal.pone.0244115)
Supplement: Attachment — Submitted filename: Reviewer suggestions for manuscript No. PLOS-D-20-29971.docx [file pone.0244115.s001.docx]

**Review Report on “Poly (alizarin red S) modified glassy carbon electrode for square wave adsorptive stripping voltammetric determination of metronidazole in tablet formulation”**

Manuscript Number: PONE-D-20-29971

The authors have given detailed information for the detection of metronidazole by glassy carbon electrode modified with electro-polymerized alizarin red S. The work lacks novelty and needs more issues to be addressed as follows:

**A. ABSTRACT**

1. Typo-errors should be adjusted along the whole manuscript.
2. SEM images of bare and unmodified GCE should be added.
3. R^2^ should be four decimal points.
4. Simple abbreviations should be used along the manuscript such as current should be Ipc, scan rate should be ν and ---------so on.
5. Recoveries % should be one decimal points for example 96.15% should be 96.2%
6. Line 20, in the presence of 100 and 150% of uric acid and ascorbic acid. Please omit these values and replace by concentrations.
7. What is meant by complex matrix? Why the authors did not quantify metronidazole in biological matrices such as urine and plasma.
8. What is meant by charge transfer controlled?

**B. INTRODUCTION**

9. Scheme 1 should be removed. The structure of metronidazole is already described in the reduction mechanism.
10. Metronidazole should be properly abbreviated as MTZ along the manuscript.
11. Thus, development of a simple, cost effective, and sensitive method for determination of MTZ in samples like tablet formulation is still vital. Please omit “like tablet formulation”.
12. The authors should add a simple paragraph about the importance of polymers in the fabrication of electrodes, and the following papers for references that force this section:

<https://doi.org/10.1016/j.msec.2017.02.092>, https://doi.org/10.1007/s00216-019-02245-8, https://doi.org/10.1002/elan.201700078, https://doi.org/10.1016/j.colsurfa.2019.01.033

13. Lines 73-75 not correct and should be corrected i.e. what is meant by glassy carbon electrode is widely used due to its biocompatibility with tissue (incorrect statement) and minimal propensity to show deteriorated response as a result of electrode fouling (not correct as the GCE itself cannot prevent fouling itself but the some modifier on its surface prevents its fouling).

**III. EXPERIMENTAL**

14. Abbreviations such as PARS and ARS should be defined at start i.e. ABSTRACT.
15. Term MET instead of metronidazole should be unified along the revised manuscript.
16. Concentration of supporting electrolyte in reference electrode should be mentioned.
17. Section 2.5, 10 mM should be replaced by amount in grams.
18. The authors should perform MET analysis in more interfering samples such as urine and plasma.

**IV. RESULTS AND DISCUSSIONS**

19. In Fig.1, to prove the stability of poly (alizarin S) on the surface of GCE, multiple CV scans should be provided on inset of Fig.1.
20. Accumulation time should be added to Fig.2.
21. Surface areas of bare and modified GCE should be calculated.
22. Scan rate should be unified along the manuscript either mV/s or mV s^-1^.
23. Figs. 5 and 6 should be merged in one Figure.
24. Line 257, R^2^ is determination coefficient **not** correlation coefficient.
25. What is the relationship between R^2^ and type of analyte transfer during electroanalysis (lines 257-260?
26. “Slope value of 0.38, which is less than the ideal value of 0.5 for plot of log Ip versus log ʋ (Fig. 9), further confirmed participation of both with adsorption as the predominant”. This is incorrect as slope near to 0.5 means diffusion controlled process while near to 1.0 means adsorption controlled. Please, use these references as a guide.

https://doi.org/10.1016/j.bios.2018.03.015, https://doi.org/10.1016/j.molliq.2018.08.105, https://doi.org/10.1016/j.bios.2019.111849

27. Fig.8, what is meant by SQRT?
28. Figs. 7, 8 and 9 should be merged in one Figure.
29. Accumulation time should be added to Fig.10.
30. Fig.11 represents calibration plot of MTZ in bulk or tablet. Please, specify.
31. Please, replace SWAdCSV by SWV.
32. Method should be compared with a reference method.
33. Why the authors tested selectivity of the electrode using only ascorbic and uric acids, although they performed their analysis if pharmaceutical formulations that include other excepients such as glucose, sucrose, Mg-stearate, maltose, starch and others. Ascorbic and uric acids are substances mainly present in biological samples. So, it is preferred to carry out analysis in biological fluid e.g. plasma
34. Figs. 12 and 13, replace SWSV by SWV.
35. Caption of Table 3, replace metronidazole with MTZ.
36. To Table 3, add samples analyzed.
37. Table 3, replace DPSV by DPV.

**V. CONCLUSION (S)**

38. Conclusion part should be shortened and contains all corrected issues raised by the reviewer.
39. Replace metronidazole by MTZ in conclusion part.
