# Supplementary material for: Poly(alizarin red S) modified glassy carbon electrode for square wave adsorptive stripping voltammetric determination of metronidazole in tablet formulation
Source: PLoS One. 2020 Dec 22;15(12):e0244115. doi: 10.1371/journal.pone.0244115 (PMC7755201; doi:10.1371/journal.pone.0244115)
Supplement: Attachment — Submitted filename: Response to comments by reviewers.doc [file pone.0244115.s002.doc]

**Response to comments by reviewers**

The authors would like to thank the reviewers (all) for their genuine and critical comments on our paper. Almost all the comments were accepted and addressed in the revised document.

**Reviewer #1**

**A. ABSTRACT**

1. Typo-errors should be adjusted along the whole manuscript.

**Ans:** The comment is addressed in the revised version.

2. SEM images of bare and unmodified GCE should be added.

**Ans:** For two things we did not add the SEM image. A) It is already reported elsewhere B) The instrument is not available around us.

3. R^2^ should be four decimal points.

**Ans:** We revised the R^2^ value to be four decimal points.

4. Simple abbreviations should be used along the manuscript such as current should be Ipc, scan rate should be ν and ---------so on.

**Ans:** It is addressed.

5. Recoveries % should be one decimal points for example 96.15% should be 96.2%

**Ans:** We did it.

6. Line 20, in the presence of 100 and 150% of uric acid and ascorbic acid. Please omit these values and replace by concentrations.

**Ans:** Accepted and addressed.

7. What is meant by complex matrix? Why the authors did not quantify metronidazole in biological matrices such as urine and plasma.

**Ans:** A) By complex matrix, it was to mean the active ingredient added with the excipients in a tablet. (B) Determination of MTZ in biological matrices using the present method will be our next task.

8. What is meant by charge transfer controlled?

**Ans:** Correct comment; it is now addressed.

**B. INTRODUCTION**

9. Scheme 1 should be removed. The structure of metronidazole is already described in the reduction mechanism.

**Ans:** We believe the reader need the structure while reading the introductory part on the functional groups, etc.

10. Metronidazole should be properly abbreviated as MTZ along the manuscript.

**Ans:** The comment is addressed throughout the document.

11. Thus, development of a simple, cost effective, and sensitive method for determination of MTZ in samples like tablet formulation is still vital. Please omit “like tablet formulation”.

**Ans:** The statement is revised.

12. The authors should add a simple paragraph about the importance of polymers in the fabrication of electrodes, and the following papers for references that force this section:

<https://doi.org/10.1016/j.msec.2017.02.092>, https://doi.org/10.1007/s00216-019-02245-8, <https://doi.org/10.1002/elan.201700078>, <https://doi.org/10.1016/j.colsurfa.2019.01.033>

**Ans:** The comment by the reviewer is correct. Most relevant two articles are sited in the revised version.

13. Lines 73-75 not correct and should be corrected i.e. what is meant by glassy carbon electrode is widely used due to its biocompatibility with tissue (incorrect statement) and minimal propensity to show deteriorated response as a result of electrode fouling (not correct as the GCE itself cannot prevent fouling itself but the some modifier on its surface prevents its fouling).

**Ans:** the comment is not accepted. The statement is about the properties of GCE **relative to metal electrodes**. So, the comment is not accepted.

**III. EXPERIMENTAL**

14. Abbreviations such as PARS and ARS should be defined at start i.e. ABSTRACT.

**Ans:** Non-standard abbreviations are removed from the abstract. Abbreviations are used in the other parts of the paper following definition.

15. Term MET instead of metronidazole should be unified along the revised manuscript.

**Ans:** MTZ is used as an abbreviation consistently.

16. Concentration of supporting electrolyte in reference electrode should be mentioned.

**Ans:** The comment is addressed.

17. Section 2.5, 10 mM should be replaced by amount in grams.

**Ans:** Thanks to the reviewer, now the mass of tablet powder is added.

18. The authors should perform MET analysis in more interfering samples such as urine and plasma.

**Ans:** The authors’ feature plan is to work on the applicability of the method for determination of MTZ in real samples recommended by the reviewer. Thanks so much.

**IV. RESULTS AND DISCUSSIONS**

19. In Fig.1, to prove the stability of poly (alizarin S) on the surface of GCE, multiple CV scans should be provided on inset of Fig.1.

**Ans:** Normally, fresh polymer modified electrode does not give a stable voltammogram just at the first successive scans. We need to scan it in a suitable solvent (electrolyte) until it gives steady state voltammogram. Thus, stability of the polymer film refers to the film stability after the steady stae voltammogram. For our case, the fabricated modified electrode was stabilized in a monomer free 0.5 M H2SO4. The voltammogram in the inset of Fig. 1 is thus just after its stabilization.

20. Accumulation time should be added to Fig.2.

**Ans:** Fig. 2 is about the response of the electrode towards {Fe(CN)6]3-/4- relative to the response of the unmodified for the same, which serves as a tool to confirm the surface modification.

21. Surface areas of bare and modified GCE should be calculated.

**Ans:** Correct comment. However, as the modifier is already reported somewhere, we failed to documente the CVs of {Fe(CN)6]3-/4- at different scan rates.

22. Scan rate should be unified along the manuscript either mV/s or mV s^-1^.

**Ans:** the comment is addressed in the revised version.

23. Figs. 5 and 6 should be merged in one Figure.

**Ans:** Done.

24. Line 257, R^2^ is determination coefficient **not** correlation coefficient.

**Ans:** Corrected.

25. What is the relationship between R^2^ and type of analyte transfer during electroanalysis (lines 257-260?

**Ans:** The R^2^ for dependence of the peak current on scan rate and square root of scan rate need to be compared to predict the type of kinetics that determined the rate of the reaction.

26. “Slope value of 0.38, which is less than the ideal value of 0.5 for plot of log Ip versus log ʋ (Fig. 9), further confirmed participation of both with adsorption as the predominant”. This is incorrect as slope near to 0.5 means diffusion controlled process while near to 1.0 means adsorption controlled. Please, use these references as a guide.

https://doi.org/10.1016/j.bios.2018.03.015, https://doi.org/10.1016/j.molliq.2018.08.105, <https://doi.org/10.1016/j.bios.2019.111849>

**Ans:** We investigated effect of scan rate on peak current and compared the dependence of the peak current on the scan rate and square root of scan rate using the respective R2 values. We found the R2 for dependence of current on scan rate greater than that on the square root. This indicated participation of both diffusion and adsorption with predominantly adsorption controlled. Further confirmation was the slop of log current vs log scan rate. A slope of 0.38 is away from the ideal value 0.5 for diffusion controlled. Lastly, we generalized that the reaction was controlled by adsorption by citing reference for it. The recommended references are relevant only for cases where the slope of plot of log(current) vs log(scan rate) is 0.5 or above. What about if the slope is below 0.5 as the case of ours?

27. Fig.8, what is meant by SQRT?

**Ans:** Corrected.

28. Figs. 7, 8 and 9 should be merged in one Figure.

**Ans:** The comment to merge the three figures is appreciated. However, as long as there is no limit, we preferred to put them separately so that the readers can understand without confusion.

29. Accumulation time should be added to Fig.10.

**Ans:** Fig. 10 (now Fig. 9) is recorded before investigation of effect of accumulation parameters (Eacc & tacc).

30. Fig.11 represents calibration plot of MTZ in bulk or tablet. Please, specify.

**Ans:** the method used is standard addition (calibration by adding standards to a tablet sample).

31. Please, replace SWAdCSV by SWV.

**Ans:** While SWV is a broad method, the method we reported is stripping, specifically adsorptive. Thus, SWAdCSV represents “square wave adsorptive cathodic stripping voltammetry”.

32. Method should be compared with a reference method.

**Ans:** That is correct. We didn’t for two reasons. A) it is unfortunate to have accessible reference method (HPLC) in our lab. B) The good thing in working with pharmaceutical formulations is, the company label can be taken as a reference value.

33. Why the authors tested selectivity of the electrode using only ascorbic and uric acids, although they performed their analysis if pharmaceutical formulations that include other excepients such as glucose, sucrose, Mg-stearate, maltose, starch and others. Ascorbic and uric acids are substances mainly present in biological samples. So, it is preferred to carry out analysis in biological fluid e.g. plasma

**Ans:** The comment is correct. However, the effect of the excipients is already checked for a tablet sample that contained the active ingredient and excipient is used. So, AA and UA are taken as potential inteferents in case when a biological sample is taken.

34. Figs. 12 and 13, replace SWSV by SWV.

**Ans:** Not accepted for the reasons under comment #11.

35. Caption of Table 3, replace metronidazole with MTZ.

**Ans:** Done.

36. To Table 3, add samples analyzed.

**Ans:** Done

37. Table 3, replace DPSV by DPV.

**Ans:** Removed.

**V. CONCLUSION (S)**

38. Conclusion part should be shortened and contains all corrected issues raised by the reviewer.

**Ans:** Done.

39. Replace metronidazole by MTZ in conclusion part.

**Ans:** Done.

**Reviewer #2:**

The results presented by the authors are important to understand a novel way to determine metroimidazole by voltammetry method using a modified electrode which is quite important. The findings are interesting. There are some minor revisions upon.
1. Please read MS carefully there are some minor grammar mistakes.

**Ans:** Revised.
2. Insert the error bars in Figure 6 and 11.

**Ans**: Done.
3. The conclusion should be concise and highlighted the obtained results.

**Ans:** Revised.

**Reviewer #3:**

The manuscript introduced a stripping voltammetric method for the determination of
metronidazole in tablet sample based on the poly(alizarin red s) modified GCE. Although the method is simple, however, the author has not pointed out the advantages of it, when compared with other electrochemical methods for the determination of metronidazole. And the following questions should be considered by the author：
1. In line 106 and 107, “working” and “reference” can not be fully expressed, it should be replaced by professional terms such as working electrode, reference electrode.

**Ans:** Done
2. The morphology of poly alizarin red material should be characterized by SEM if possible.

Ans: The modified electrode is already reported previously.
3. Some typing error should be corrected.

**Ans:** Revised.
4. Table 2 should be represented by a three line table.

**Ans:** Done.
5. For the explanation of inner probes, it is better to compare the electrochemical behavior of outer probes such as ruthenium ammonia. In addition, some References is need to explanation the inner probes. For example, the article of Richard L. McCreery, <https://pubs.acs.org/doi/abs/10.1021/ac960492r>.

**Ans:** The comment is not clear.
6. Error bars should be added to the calibration curve of Ic to c.

**Ans:** Done.
