# Supplementary material for: Poly(alizarin red S) modified glassy carbon electrode for square wave adsorptive stripping voltammetric determination of metronidazole in tablet formulation
Source: PLoS One. 2020 Dec 22;15(12):e0244115. doi: 10.1371/journal.pone.0244115 (PMC7755201; doi:10.1371/journal.pone.0244115)
Supplement: Attachment — Submitted filename: Reviewer suggestions for manuscript No. PLOS-D-20-29971R1.docx [file pone.0244115.s003.docx]

**Review Report on “Poly (alizarin red S) modified glassy carbon electrode for square wave adsorptive stripping voltammetric determination of metronidazole in tablet formulation”**

Manuscript Number: PONE-D-20-29971 R1

The authors have given detailed information for the detection of metronidazole by glassy carbon electrode modified with electro-polymerized alizarin red S. The work can be accepted after addressing the following:

In General: the abbreviations along the revised version should be used properly such as metronidazole should be MTZ, cyclic voltammetry should be CV and square wave voltammetry----etc.

**A. ABSTRACT**

1. Lines 9-10, with three-folds of current enhancement at the modified GCE. Compared to what?
2. Lines 13-15, this sentence should be adjusted.
3. Lines 20 and 21, concentration units of ascorbic and uric acids should be added.
4. Line 25, with complex matrix should be removed. Most tablets contain electro-inactive species, while biological samples contain more interfering species.

**B. INTRODUCTION**

5. Line 46, scheme1 should be removed.
6. The novelty of the manuscript is still unclear. The authors should force the readers’ attention via stressing on the novelty of the method compared to others.

**B. EXPERIMENTAL**

7. Internal diameter of GCE should be added.
8. Line 126, 5 mM should be 5.0 mM.
9. Line 127, 1mM should be 1.0 mM.

**B. RESULTS AND DISCUSSIONS**

10. Calculation of effective surface areas of GCE and modified GCE should be added.
11. Reproducibility and stability of PARS/GCE should be addressed.
12. To show accuracy of the method, it should be compared with reported method.
