# Supplementary material for: Poly(alizarin red S) modified glassy carbon electrode for square wave adsorptive stripping voltammetric determination of metronidazole in tablet formulation
Source: PLoS One. 2020 Dec 22;15(12):e0244115. doi: 10.1371/journal.pone.0244115 (PMC7755201; doi:10.1371/journal.pone.0244115)
Supplement: Attachment — Submitted filename: Response to reviewers comments.doc [file pone.0244115.s004.doc]

**Response to comments by reviewers**

Firstly, we authors appreciate the reviewers for their critical comments. Below is our point-by-point response to comments by the reviewers.

**Reviewer #1**

General comment:
In General: the abbreviations along the revised version should be used properly such as metronidazole should be MTZ, cyclic voltammetry should be CV and square wave voltammetry----etc.

Response

- Thanks to the reviewer, we have revised the document addressing the comment.

ABSTRACT

1. Lines 9-10, with three-folds of current enhancement at the modified GCE. Compared to what?

Answer: addressed in the revised document.

2. Lines 13-15, this sentence should be adjusted.

Answer: the sentence is rewritten.

3. Lines 20 and 21, concentration units of ascorbic and uric acids should be added.

Answer: common unit is added.

4. Line 25, with complex matrix should be removed. Most tablets contain electro-inactive species, while biological samples contain more interfering species.

Answer: In electrochemistry, interferent does not only refer to an electroactive species but also to any form of substance that may alter the result may be by undergoing a reaction with the analyte of interest or by competing the potential space of the analyte. Thus, by a complex matrix in our context refers to any including the excipient. Thus, the comment is not accepted.

INTRODUCTION

5. Line 46, scheme1 should be removed.

Answer: the reason why it is customary to put the structure of the analyte in the document is it helps the reader to predict (ask himself) the possible reaction sites and reaction type the analyte could undergo. Therefore, the authors are not convinced why to remove the scheme.

6. The novelty of the manuscript is still unclear. The authors should force the readers’ attention via stressing on the novelty of the method compared to others.

Answer: Thanking the reviewer for his/her critical comment, we have tried to modify the last statement of the introduction part addressing the comment.

EXPERIMENTAL

7. Internal diameter of GCE should be added.

Answer: the internal diameter of the GCE used in this study is already mentioned under section 2.3.

8. Line 126, 5 mM should be 5.0 mM.

Answer: Addressed.

9. Line 127, 1mM should be 1.0 mM.

Answer: Addressed.

RESULTS AND DISCUSSIONS

10. Calculation of effective surface areas of GCE and modified GCE should be added.

Answer: The comment to determine effective surface area among the two possible contributors for catalytic activity (conductivity, and electrode effective surface area) is highly appreciated. Although we have shown the conductivity improvement of the modified electrode, we failed to have data of the effect of scan rate on [Fe(CN)6]^3-/4-^. Appreciating the comment, we couldn’t include the data in the revised document.

11. Reproducibility and stability of PARS/GCE should be addressed.

Answer: For the ***absolute completeness*** of our work, we should have validated it using parameters including the reproducibility and stability of the modified electrode. Appreciating the reviewer’s comment, we failed to do it for we are out of lab for CORONA reason.

12. To show accuracy of the method, it should be compared with reported method.

Answer: the performance of our method is compared with the nominal tablet labeled value and further validated by the recovery (spike and interference) results.

**Reviewer #3**

1. In line 110 in the PONE-D-20-29971-R1, “Ag/AgCl (3 M KCl) as reference” should be replaced by professional terms such as reference electrode.

Answer: To avoid confusions between the different reference electrode like calomel, standard hydrogen electrode, Ag/AgCl (saturated,…), it is advisable to put the specific electrode used.

2. Table 2 still needs to be modified and it should be on the same page. The form of the table 2 is as shown in Table 3.

Answer: Thanking the comment by the reviewer, we have polished the table (removed the raw lines).

3. Incorrect sentence and typing error still exist. Please check it carefully and correct it. For example, “0.98830 and 0.97398” in line 261, “[32 33.” in line 264 in the PONE-D-20-29971-R1.

Answer: we have revised the document addressing typographic errors including what the reviewer as indicated. However, the “0.98830” and “0.97398” which are the calculated determination coefficients are important values based on which we proposed an adsorption reaction kinetics.

4. Some superscripts and subscripts should be carefully checked and revised.

Answer: We have revised the document addressing such errors.
